# Carbazole Derivatives as Potential Antimicrobial Agents

**DOI:** 10.3390/molecules27196575

**Published:** 2022-10-04

**Authors:** Siddappa A. Patil, Shivaputra A. Patil, Ever A. Ble-González, Stephen R. Isbel, Sydney M. Hampton, Alejandro Bugarin

**Affiliations:** 1 Department of Chemistry & Physics, Florida Gulf Coast University, 10501 FGCU Boulevard South, Fort Myers, FL 33965, USA; 2 Pharmaceutical Sciences Department, College of Pharmacy, Rosalind Franklin University of Medicine and Science, 3333 Green Bay Road, North Chicago, IL 60064, USA

**Keywords:** carbazole derivatives, antimicrobial, antifungal, antibacterial, drug resistance

## Abstract

Microbial infection is a leading cause of death worldwide, resulting in around 1.2 million deaths annually. Due to this, medicinal chemists are continuously searching for new or improved alternatives to combat microbial infections. Among many nitrogen-containing heterocycles, carbazole derivatives have shown significant biological activities, of which its antimicrobial and antifungal activities are the most studied. In this review, miscellaneous carbazole derivatives and their antimicrobial activity are discussed (articles published from 1999 to 2022).

## 1. Introduction

Drug resistance due to overpopulation, increased use, and various other reasons is one of the key factors for most deaths caused by microbial diseases [1,2]. The World Health Organization (WHO) recently estimated that global death caused by antimicrobial-resistant pathogens will reach 10 million people per year by 2050 [3]. There are several critical factors that are responsible for drug resistance, such as a high rate of mutations, ineffectiveness towards available clinical agents, lack of awareness about the overuse of antibiotic drugs that are available over the counter, and, more significantly, the non-availability of newer drugs due to the lack of drug discovery and development strategies; these factors are responsible for drug resistance in microbial diseases [4,5]. Currently, clinically effective treatments with molecular pharmacophores such as macrolides, quinolones, tetracycline, glycopeptide, and cephalosporins have encountered tremendous challenges, including increased resistance due to the above-mentioned issues [6,7,8,9,10,11,12,13,14]. Microorganisms in areas of high drug use, such as hospitals, for example, continue to mutate rapidly and can spread among patients, becoming more resistant to current treatments [10]. These resistant microorganisms will continue to pose unforeseen health problems globally. Drug resistance is continually increasing due to many factors and does not appear to be slowing, making this issue of the utmost importance. Therefore, the antimicrobial research community must continually work together to design and develop new and safer small molecule drugs to overcome amassing drug resistance along with toxicity problems. The medicinal chemistry community works systematically to identify novel pharmacophores or new molecular entities (NMEs) to overcome these problems. Then, researchers combine medicinal, computational, and synthetic chemistry knowledge along with drug resistance to successfully develop efficacious new drug candidates with novel pharmacophoric functionalities.

**Figure 1:**
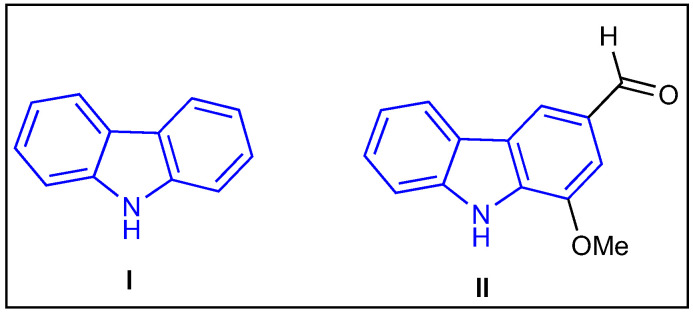
Structure of 9*H*-carbazole (**I**) and its analog murrayanine (**II**).

Carbazole is one of the privileged nitrogen-containing heterocyclic pharmacophores in medicinal chemistry due to its attractive drug-like properties and outstanding biological activities. Carbazole (**I**) was first isolated by Graebe and Glazer in 1872 from coal tar (Figure 1) [15]. The carbazole analog, murrayanine (1-methoxy-3-formylcarbazole) (**II**), was isolated from the stem-back of *Murraya koenigii* Spreng by Chakraborty, Barman, and Bose in 1965 (Figure 1) [16]. Since then, carbazole analogs have attracted both medicinal chemists and biologists alike due to their ever-growing pharmacological activities [17,18,19]. Thus, carbazole has become a privileged scaffold to access natural and unnatural biologically active molecules [20].

**Figure 2:**
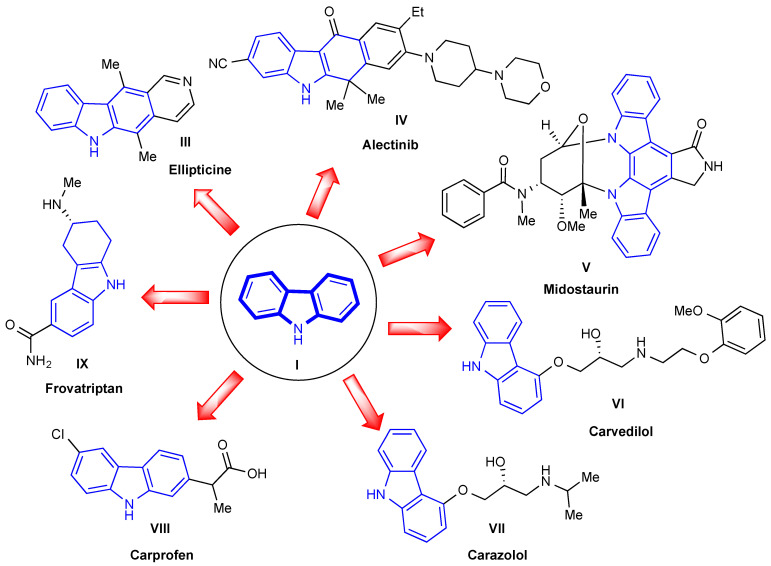
Structures of notable carbazole ring containing approved drugs.

Therapeutically important natural carbazoles have inspired synthetic medicinal chemists to design and develop novel (semi)synthetic carbazole derivatives. The carbazole moiety is present in several important commercially available drug molecules such as ellipticine (**III**), alectinib (**IV**), midostaurin (**V**), carvedilol (**VI**), carazolol (**VII**), carprofen (**VIII**), and frovatriptan (**IX**) (Figure 2). The naturally occurring alkaloid ellipticine (**III**) was first discovered in 1959 as an anticancer agent. It was extracted from the leaves of *Ochrosia elliptica* and is considered to be one of the initial leading therapeutic carbazole analogs for the treatment of cancer [21]. Alectinib (**IV**) was approved by the FDA in 2015 for the treatment of advanced non-small cell lung cancer (NSCLC) [22]. Midostaurin (**V**) was permitted by the FDA in 2017 for the treatment of newly diagnosed acute myeloid leukemia (AML) and for advanced systemic mastocytosis (SM) [23,24].

Carvedilol (**VI**) was approved in 1995 to treat high blood pressure and congestive heart failure (CHF) [25]. Carazolol (**VII**) is a truncated analog of carvedilol and was approved to treat cardiovascular disorders in non-human mammals [26]. Carprofen (**VIII**) is a non-steroidal anti-inflammatory drug (NSAID) and has been used to relieve pain and inflammation in animals [27]. The 5-hydroxytryptamine (5-HT) receptor agonist, frovatriptan (**IX**), has been approved for the acute treatment of moderate to severe migraines in humans [28]. Even though there exist several carbazole containing-drugs that have been approved to treat various diseases, their full biological potential has yet to be exploited, and therefore, breakthroughs remain to be discovered. This has generated renewed interest in discovering and developing new carbazole analogs with improved efficacy and reduced toxicity for various diseases, including microbial and fungal diseases. The present review discusses the synthesis and biological activities of carbazole analogs as antimicrobial agents.

## 2. Antibacterial and Antifungal Activities of Carbazole Derivatives

**Figure 3:**
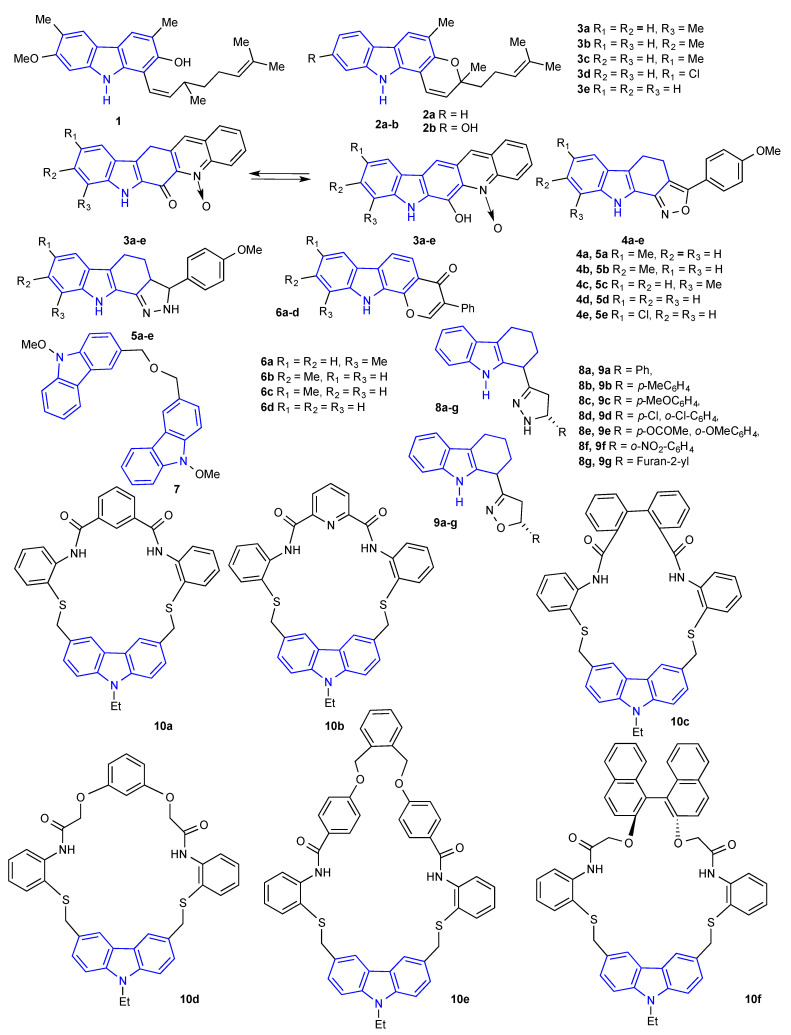
Structures of the reported carbazole derivatives from **1999 to 2009**.

Compounds containing the carbazole moiety are a favorable class of agents against numerous diseases and are conceivably useful in clinical studies. In 1999, Nair et al. separated carbazole alkaloids (**1** and **2a-b**) (Figure 3) from *Murraya koenigii* leaves. Carbazole alkaloid **1** demonstrated high activity towards *C*. *kruseii*, *C*. *parapsilasis*, *E*. *coli*, *S*. *aureus*, and *S*. *pyogenes* species, while carbazole alkaloid **2a** exhibited low activity against these test species. Carbazole alkaloids **2b** and **1** gave minimum inhibitory concentration (MIC) against *S*. *aureus,* and *S*. *pyogenes* at 25 µg/mL [29]. In 2003, Prasad et al. reported the one-pot preparation and evaluation of carbazole derivatives (**3a-e**) (Figure 3) as antimicrobial agents. The chloro-substituted derivative **3d** exhibited outstanding antimicrobial activity against *E. coli*, *S. aureus*, *P. aeruginosa,* and *B. subtilis,* whereas carbazole derivatives **3a** and **3c** displayed slightly lower activity against all the bacterial species tested. Carbazole derivative **3d** demonstrated excellent antifungal activity towards all the fungi screened in comparison with the standard drug carbendazim. This high activity might be due to the presence of chloro and *N*-oxide groups in the compound [30]. One year later, in 2004, Prasad et al. also reported the antimicrobial activities of isoxazolo- and pyrazolino-annulated carbazoles (**4a-e** and **5a-e**) (Figure 3) against certain pathogenic bacterial and fungal species. The antimicrobial activity of all the carbazole derivatives (**4a-e** and **5a-e**) has been compared with the standard fungicide bavistin and the bactericide streptomycin. Almost all the carbazole derivatives (**4a-e** and **5a-e**) demonstrated exceptional activity against both the organisms of bacteria and fungi [31]. In another report, Prasad et al. also investigated the synthesis and characterization of carbazole derivatives (**6a-d**) (Figure 3). All the compounds (**6a-d**) were screened for their in vitro antimicrobial activities towards *A. hydrophila*, *P. aeruginosa*, *B. subtilis*, *A. niger,* and *Fusarium* species through the disc diffusion method. Among the compounds screened, **6d** was found to have higher activity compared to the remaining compounds [32]. Extraction, characterization, and antimicrobial activity of carbazole alkaloid (**7**) (Figure 3) from *Murraya koenigii* was explored from Rahman et al. The structure of the carbazole alkaloid (**7**) was confirmed by various spectroscopic techniques. The carbazole alkaloid (**7**) was then tested for antimicrobial activities by a microdilution technique and exhibited strong antimicrobial activity towards *E. coli, P. vulgaris, A. niger,* and *C. albicans* species [33].

Antimicrobial properties of another series of carbazole derivatives (**8a-g** and **9a-g**) (Figure 3) were described by Surendiran et al. at a concentration of 25 µg/mL using ciprofloxacin and ketoconazole as a standard. Carbazole derivatives (**8a**), (**8d**), (**9c**), and (**9d**), having methoxy groups and chloro groups in heterocyclic moieties, demonstrated moderate to good antibacterial activities. Carbazole derivatives (**8e**) and (**9e**), having acetyloxy and methoxy groups, displayed more pronounced activities than the other tested compounds. Compounds (**9c**) and (**9d**) exhibited excellent antifungal activity against *F. porum* and *A. macrospore* species, whereas compounds (**8b**) and (**9b**) displayed moderate activity [34].

**Figure 4:**
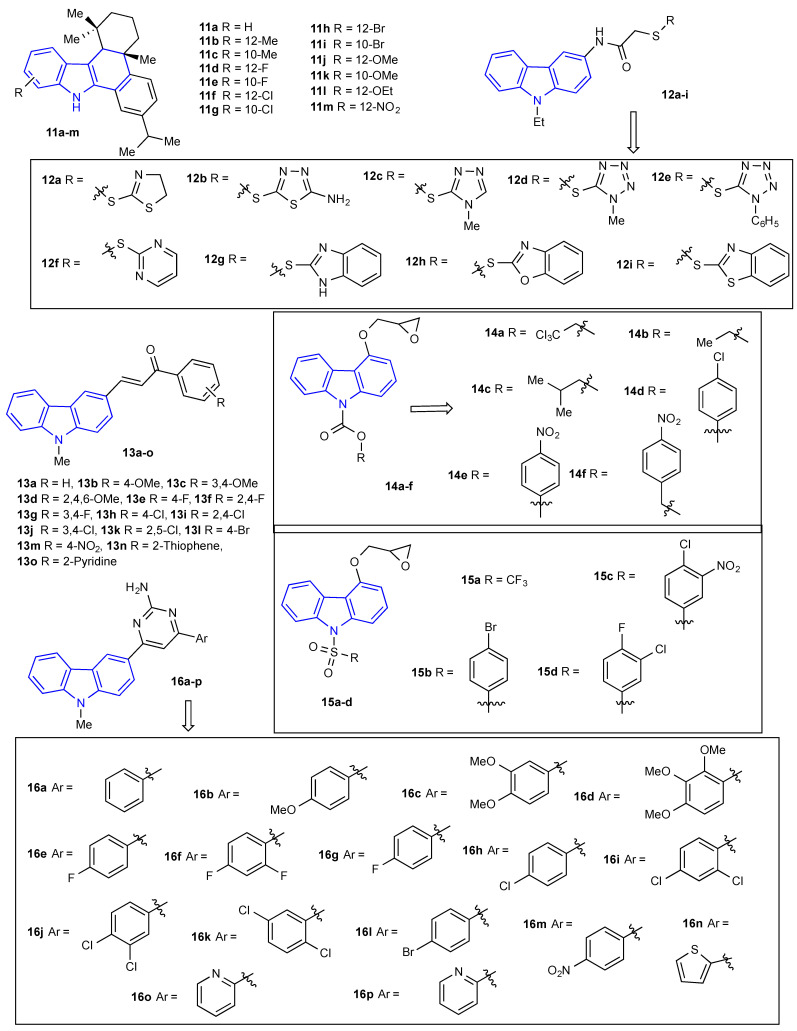
Structures of the reported carbazole derivatives from **2010 to 2013**.

Macrocyclic compounds have also received significant attention due to their biological activities. Rajakumar et al. evaluated the antimicrobial activity of a series of sulfur and oxygen linkages containing carbazole-based macrocyclic diamides (**10a-f**) (Figure 3). The MIC of carbazole amides (**10a-f**) towards bacterial species was determined to have a concentration range between 5 and 80 µg/mL. The macrocyclic diamide **10f** displayed significant antibacterial activity towards all the screened bacterial species when compared to the rest of the macrocyclic diamides and standard antibiotic tetracycline at low concentrations ranging from 5 to 10 µg/mL. In contrast, MICs of the macrocyclic diamides (**10a-f**) towards fungal species were determined to have a concentration range between 10 and 45 µg/mL. Among the macrocyclic diamides (**10a-f**) screened, macrocyclic diamide **10f** considerably inhibited all four of the fungal species with a concentration range between 10 to 20 µg/mL when compared to the rest of the macrocyclic diamides and standard fungicide, carbendazim [35]. Gu et al. reported the preparation and antimicrobial activities of novel 1H-dibenzo[a,c]carbazoles by dehydroabietic acid. All the prepared carbazoles (**11a-m**) (Figure 4) were examined for their activity against four bacteria and three fungal species. Among the screened carbazoles, **11d**-**f** and **11m** revealed strong (1.9–7.8 µg/mL) antibacterial activities, whereas carbazoles **11e** and **11m** displayed moderate antifungal activities. Principally, **11d** demonstrated stronger (1.9 µg/mL) antibacterial activity towards *B. subtilis* when compared to the current standard drug amikacin [36]. In modern drug synthesis, an essential constituent of the search for new leads is the preparation of the library of new molecules. Synthesis of new carbazole derivatives and an evaluation of their antimicrobial activity were investigated by Kaplancikli et al. Antimicrobial activities of all the compounds were established using the microbroth dilution method with chloramphenicol and ketoconazole as the standard drug. Almost all compounds (**12a-i**) (Figure 4) were effective towards *C. albicans*. When compared with ketoconazole, **12c** and **12d in particular** displayed similar activity, and **12a, 12b**, **12e**, **12f** revealed moderate activity towards *C. albicans*. Comparable results were obtained from *C. glabrata*. The antibacterial activity revealed that carbazole derivatives (**12a-i**) exhibited moderate or slight activity with MIC values within the range of 100->400 μg/mL towards all the bacterial species. By comparing their MIC values with the standard drug chloramphenicol, the carbazole derivatives (**12a-i**) were less active towards *E. coli* and *S. aureus*. Conversely, the carbazole derivatives (**12a-i**) exhibited comparable or better activities towards *P. aeruginosa* than did chloramphenicol [37]. Bandgar et al. explored the antimicrobial activities of a series of novel carbazole chalcones (**13a-o**) (Figure 4). Carbazole chalcones **13a**, **13e** and **13m** revealed good antifungal activity towards all three fungal growths. Compounds **13b**, **13g** and **13h** exhibited good antibacterial activity against *P. vulgaris* and *E. coli* selectively, and compounds **13c** and **13o** were active against *S. aureus.* Additionally, the antifungal screening data revealed that compounds **13h** and **13m** exhibited good antifungal activity, while the remaining compounds were inactive towards *C. albicans* [38]. Synthesis and spectral characterization of sulfonamide and carbamate derivatives of 4-(oxiran-2-ylmethoxy)-9*H*carbazole (**14a-f** and **15a-d**) (Figure 4) were described by Raju et al. All the synthesized compounds (**14a-f** and **15a-d**) were investigated regarding their in vitro antibacterial (*S. aureus*, *B. subtilis,* and *E. coli*) and antifungal (*F. oxysporum*, *C. albicans,* and *A. niger*) activities through the agar well diffusion technique. All the compounds (**14a-f** and **15a-d**) revealed moderate to strong antimicrobial activities at a concentration of 200 μg/mL, and the results were comparable to the standard drugs ciprofloxacin and fluconazole [39].

The pyrimidine moiety is one of the most noticeable structures found in the nucleic acid. In 2012, Bandgar et al. examined the antimicrobial activity of a series of new carbazole substituted aminopyrimidines (**16a-p**) (Figure 4) using the disk diffusion method. Carbazole derivatives **16c**, **16g**, **16j**, and **16o** displayed modest activity towards all the selected bacterial species at a concentration of 1 mg/mL as compared to the standard drug tetracycline. Carbazole derivative **16o** exhibited comparable activity to that of the standard and towards *B. subtilis*, *S. aureus* and *S. flexenari.* On the other hand, compounds **16b**, **16c**, **16m**, and **16o** exhibited decent activity against certain fungal organisms at a concentration of 1 mg/mL as compared to the standard drug nystatin. Carbazole derivatives **16m** and **16o** displayed comparable activity to that of the standard and towards *C. albicans* and *A. niger* [40].

**Figure 5:**
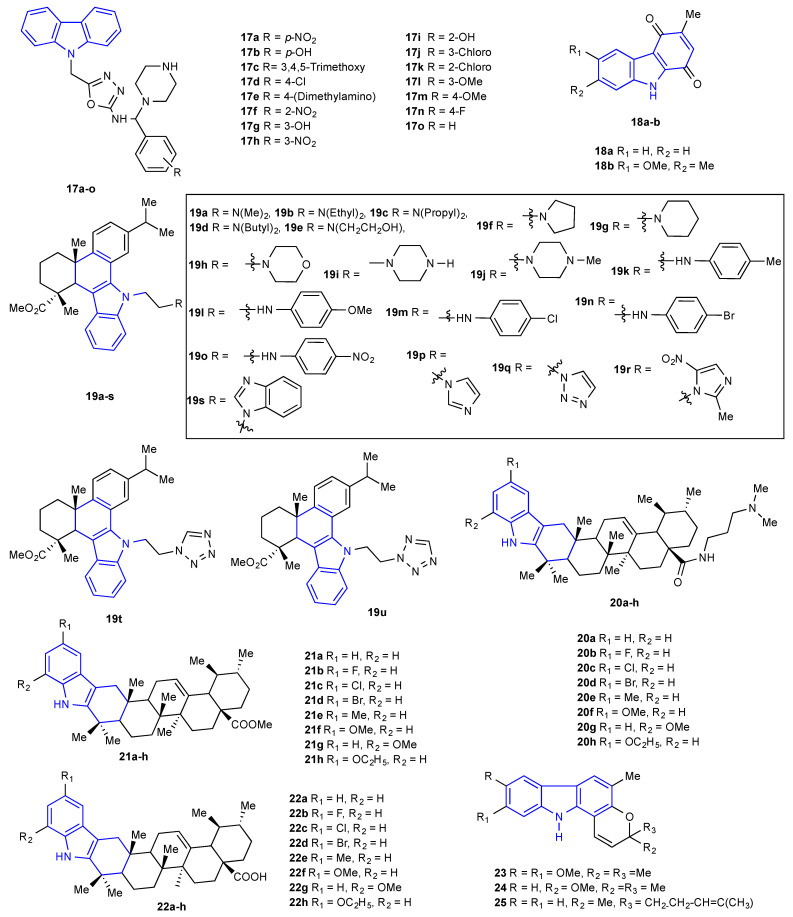
Structures of the reported carbazole derivatives from **2014 to 2015**.

Several heteroannulated carbazole compounds have drawn great attention due to their natural occurrence and their comprehensive spectrum of antimicrobial activity. In 2014, Sharma et al. examined a series of new 5-[(9*H*-carbazol-9-yl)methyl]-*N*-[(substituted phenyl)(piperazin-1-yl)methyl]-1,3,4-oxadiazol-2-amines derivatives (**17a**–**o**) that were synthesized and structurally characterized by various spectroscopic methods. All the synthesized carbazole derivatives (**17a**–**o**) (Figure 5) were screened for their antimicrobial activities from the disc diffusion technique. Among them, **17a**, **17d**, **17e**, and **17n** displayed substantial antimicrobial activity at a concentration of 50 μg/mL, and the results are comparable with standard drugs such as ciprofloxacin and fluconazole [41].

Saha et al. reported the antibacterial activity of compounds **18a**-**b** (Figure 5) against Gram-positive (*B. subtilis* and *S. aureus*) and Gram-negative (*E. coli* and *Pseudomonas* sp.) bacteria. From the data, it is confirmed that compound **18a** demonstrated higher activity towards *S. aureus* than *E. coli,* whereas compound **18b** exhibited modest activity against *S. aureus* only. As compounds **18a**-**b** have shown high activity towards *S. aureus*, the authors executed an experiment to determine the MIC of compounds **18a**-**b** towards *S. aureus,* and the results exhibit that both the compounds **18a**-**b** have an MIC value of 50 µg/mL against *S. aureus* [42]. From dehydroabietic acid, a series of new *N*-substituted 1H-dibenzo[a,c]carbazole derivatives (**19a-u**) (Figure 5) were prepared and structurally confirmed through spectral data by Gu et al. Furthermore, all the prepared carbazole compounds were screened for their antibacterial and antifungal activities against four bacteria (*B. subtilis*, *S. aureus*, *E. coli*, and *P. fluorescens*) and three fungi (*C. albicans*, *C. tropicalis*, and *A. niger*) from the serial dilution technique. Few of the prepared carbazole compounds displayed prominent antimicrobial activity towards the tested species, with MIC values ranging from 0.9 to 15.6 µg/mL. Among the carbazole compounds, **19j** and **19r** displayed effective inhibitory activity comparable to the standard drugs amikacin and ketoconazole [43]. Gu et al. reported the design, synthesis, and in vitro antimicrobial activities of the new carbazole derivatives (**20a-h**, **21a-h**, and **22a-h**) (Figure 5) of ursolic acid. All the prepared carbazole derivatives were assessed for their antimicrobial activity towards four bacterial and three fungal strains utilizing the serial dilution method [44]. Carbazole derivatives **20a-f**, **21a**, **21b**, **22a**, and **22b** demonstrated substantial antibacterial activity towards at least one of the tested bacteria with MIC values of 3.9–15.6 μg/mL. Specifically, carbazole derivative **20b** demonstrated pronounced antibacterial activity against *B. subtilis* with an MIC value of 3.9 μg/mL, near that of the positive control. Furthermore, the activities of these and other carbazole derivatives were closely pertinent to their structural properties [45,46]. Ochung et al. isolated three carbazole alkaloids, koenidine (**23**), koenimbine (**24**) and mohanimbine (**25**), from the stem bark of *Alysicarpus ovalifolius* through different solvents such as *n*-hexane, CH_2_Cl_2_, and MeOH, respectively [47]. All three of the isolated compounds (**23–25**) (Figure 5) were subjected to in vitro antimicrobial activities against yeast-like and filamentous fungi as well as some Gram-positive and Gram-negative bacteria utilizing the disc diffusion method. Dichloromethane extract showed the strongest activity towards *C. albicans* and *S. aureus* with zones of inhibition 13.2 ± 0.1 and 15.3 ± 0.1 mm in diameter, respectively, compared to the standard drugs fluconazole and amoxicillin, with zones of inhibition of 17.3 ± 0.2 and 19.5 ± 0.1 mm. Particularly, compound (**25**) showed the strongest inhibition towards *C. albicans* and *S. aureus* with zones of inhibition of 14.5 ± 0.1 and 13.8 ± 0.1 mm, respectively.

**Figure 6:**
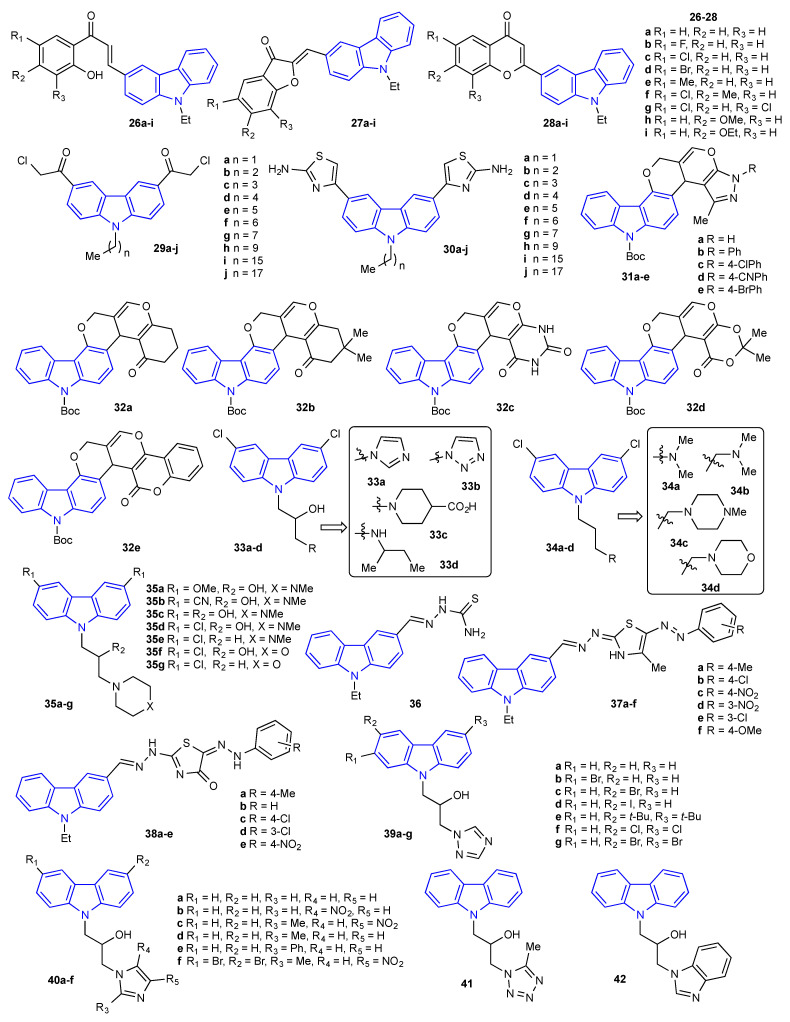
Structures of the reported carbazole derivatives from **2016 to 2018**.

In 2016, Ashok et al. reported a microwave-assisted synthesis of 27 different carbazole derivatives (9 chalcones (**26a-i**), 9 aurones (**27a-i**), and 9 flavones (**28a-i**)) (Figure 6). This library of compounds was subjected to in vitro biological studies. Two Gram-positive bacterial strains, *S. aureus* and *B. subtilis*, two Gram-negative strains, *K. pneumonia* and *E. coli*, and three fungal strains, *F. oxysporum*, *A. niger*, and *A. flavus,* were assessed. Although all compounds showed moderate activity against all compounds tested, none of them exhibited higher activity than the standards used: ciprofloxacin (for bacteria) and amphotericin-B and clotrimazole (for fungi) [48]. Additionally, in 2016, Addla et al. reported two large sets of (*N*)-alkyl carbazole derivatives (**29a-j** and **30a-j**). As depicted in Figure 6, compounds **29a-j** are alpha-chloro ketone derivatives, while adducts **30a-j** are aminothiazoles. All compounds were evaluated against four Gram-positive bacteria (*S. aureus* (ATCC259223 and N315), *B. subtilis*, and *M. luteus)*, four Gram-negative bacteria (*P. aeruginosa*, *E. typhosa*, *B. proteus*, and *E. coli)*, and five fungal strains (*C. utilis, A. favus, B. yeast, C. albicans*, and *C. mycoderma*) using the standard two-fold serial dilution method [49]. After screening, only compound **30f** showed superior growth inhibition against MRSA (N315) with an MIC value of 4 μg/mL, whereas the reference drug chloromycin gave a MIC value of 64 μg/mL, and norfloxacin gave 8 μg/mL. Similarly, Narayana et al. reported a neat synthesis and antimicrobial activity of 10 new pyrano [3,2-*c*]carbazole derivatives (**31a-e**, and **32a-e**) [50]. In addition to the antiproliferative activity of these 10 adducts, the MIC of each product was obtained for three Gram-positive bacteria, *B. subtilis, S. aureus,* and *S. epidermidis,* and three Gram-negative bacteria, *P. aeruginosa*, *K. pneumonia*, and *E. coli,* with penicillin and streptomycin used as the standard drugs. Most of the compounds showed poor antibacterial activity, with only a couple showing moderate to good activity against *P. aeruginosa* and *S. aureus*. Carbazole **32b** exhibited good activity against *P. aeruginosa* with an MIC value of 9.37 μg/mL, which is superior in comparison to penicillin (MIC = 12.5 μg/ML) [50]. Winther et al. synthesized 15 *N*-substituted carbazole derivatives (**33a-d**, **34a-d**, and **35a-g**), as depicted in Figure 6. As others have done, the new adducts were screened against seven fungal strains, *S. cerevisiae*, *C. albicans*, *C. krusei*, *C. glabrata* (ATCC 90030), *C. glabrata* (Cg003), *A. flavus*, and *A. fumigatus* [51]. The fungal growth inhibition MIC was reported using amphotericin-B as the standard drug. Of the compounds evaluated, adduct **33d** was unquestionably the most potent antifungal carbazole derivative, with MIC values ranging from 1.2 μM (*S. cerevisiae*) to 12 μM (*A. flavus*), which was comparable to that detected by amphotericin-B at 1 μM. In 2018, Farghaly et al. prepared several thiazole-containing carbazoles through microwave-assisted synthesis [52]. The synthesis started from 9-ethyl-9*H*-carbazole-3-carbaldehyde and thiosemicarbazide to produce intermediate **36**, which was then reacted with different hydrazonyl chlorides to afford carbazole derivatives **37a-f** and **38a-e** (Figure 6). All the compounds were screened against four Gram-positive bacteria, i.e., *B. subtilis*, *S. aureus*, *S*. *epidermidis*, and *S. pyogenes*, three Gram-negative bacteria, namely, *P. aeruginosa*, *K. pneumonia*, and *S. typhimurium,* and two fungi, *A. niger* and *G. candidum.* The antimicrobial data revealed that compound **38b** was the most reactive across the board. For instance, the MIC value (0.49 μg/mL) for compound **38b** against *A. niger* was superior to the standard drug amphotericin B (MIC = 0.98 μg/mL). Figure 6 also includes a set of carbazole derivatives reported by Zhang et al. Compounds **39a-g**, **40a-f**, and **41–42** were prepared following a 3-step synthetic protocol starting with commercially available carbazoles and azoles [53]. All adducts were screened against five Gram-positive bacteria (*S. aureus,* methicillin-resistant *S. aureus*, *E. faecalis*, *S. aureus* (ATCC 25923), and *S. aureus* (ATCC 29213)), six Gram-negative bacteria (*K. pneumonia*, *E. coli, A. baumannii*, *P. aeruginosa*, *P. aeruginosa* (ATCC 27853)*,* and *E. coli* (ATCC 25922)), and five fungal strains (*C. albicans*, *C. tropicalis*, *A. fumigatus*, *C. albicans* (ATCC 90023), and *C. parapsilosis* (ATCC 22019)). All adducts showed weak to modest activity again all the tested microorganisms, with the exception of compound **39f**. This compound was shown to inhibit the growth of *E. faecalis*, *S. aureus* (ATCC 29213), and *C. parapsilosis* with MIC values of 2, 4, and 4 μg/mL, respectively. These values demonstrate the superiority of this compound’s usefulness in comparison to standard drugs such as norfloxacin, which produces MIC values of 4 and 8 μg/mL with the same bacteria. Additionally, compound **39f** demonstrated an equally promising MIC value for *C. parapsilosis* as the standard drug fluconazole.

**Figure 7:**
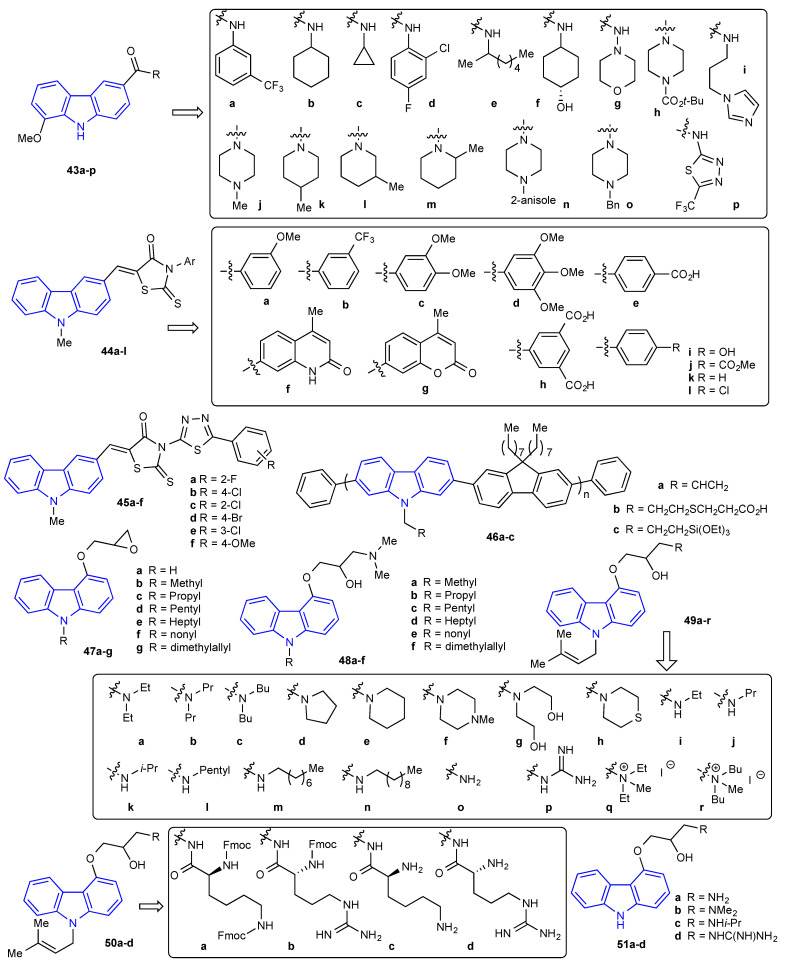
Structures of the reported carbazole derivatives from **2019 to 2020**.

In 2020, Karpoormath et al. reported a relatively large library of carbazole hybrids (**43a-p**, **44a-l**, and **45a-f**) (Figure 7). The new compounds were evaluated for their antimicrobial activity against four bacteria (*S. aureus*, *B. subtilis*, *E. coli,* and *P. aeruginosa*) and four fungal (*C. albicans*, *C. neoformans*, *C. tropicalis*, and *A. niger*) strains [54]. Among the evaluated adducts, **44g** was the most active compound with an MIC value of 3.125 μg/mL against *C. neoformans* and 1.56 μg/mL against *S. aureus*. The same year, Baycan prepared three carbazole-fluorene polymers (**46a-c**). In addition to the standard studies for a polymer (optical, electrochemical, and thermal properties), together with the surface morphology, a small antimicrobial study was reported, this time using three bacteria (*S. aureus* (ATCC 6538), *E. coli* (ATCC 1301)*,* and *E. coli* (ATCC 25922)) and one fungal (*C. albicans* (ATCC 10231)) strain. Gentamicin and cycloheximide were used as the positive controls for bacterial and fungal tests, respectively. Unfortunately, due to poor solubility, weak antimicrobial activity was observed [55]. Liu et al. synthesized a collection containing 39 compounds consisting of *N*-alkylated carbazoles with substitutions on position 4 of the carbazole (**47a-g**, **48a-f**, **49a-r**, **50a-d**, and **51a-d**) as depicted in Figure 7 [56]. With these adducts, an antibacterial study was performed using three *S. aureus* strains (ATCC 29213*,* MRSA N315*,* and MRSA NCTC10442). Of all compounds tested, guanidine-containing carbazole derivative **49p** arose as the lead compound. It exhibited excellent antibacterial activity with MIC values in the range of 0.78–1.56 μg/mL, comparable to that of the standard drug vancomycin.

**Figure 8:**
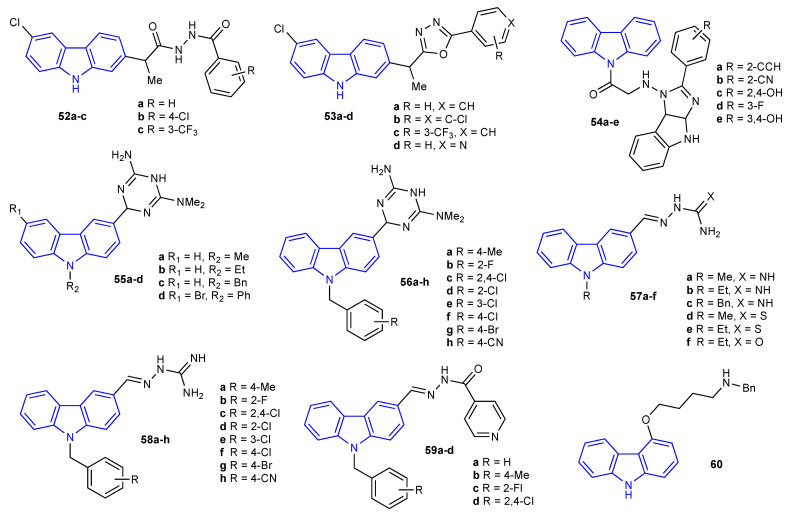
Structures of the reported carbazole derivatives from **2020 to 2022**.

In 2020, Bordei and coworkers reported the synthesis and antimicrobial activity of seven carbazole derivatives bearing either acylhydrazides (**52a-c**) or oxadiazoles (**53a-d**), as depicted in Figure 8. These seven adducts were screened against a panel of Gram-positive strains (*S. aureus* (ATCC 25923) and *E. faecalis* (ATCC 292121))*,* Gram-negative strains (*E. coli* (ATCC 25922) and *P. aeruginosa* (ATCC 27853)) and one fungus (*C. albicans* (ATCC 90029)). The MIC values were obtained using the microdilution method in liquid Mueller Hinton medium. All microorganisms showed susceptibility to the tested compounds, with **52a** displaying an MIC value of 1.25 μg/mL against *E. coli*, and **53c** displaying an MIC value of 0.625 μg/mL against *C. albicans* [57]. In 2021, Hegden et al. reported five carbazole derivatives (**54a-e**), of which the described structure is questionable (Figure 8), and their bioactivity against four microorganisms (*S. aureus*, *E. coli*, *C. albicans,* and *A. niger*). Unfortunately, none of the adducts showed better activity than the standard drugs tetracycline and griseofulvin [58]. Another large set of carbazoles containing a common miscellaneous substituent in position 3 of the carbazole backbone (**55a-d**, **56a-h**, **57a-f**, **58a-h**, and **59a-d**) was prepared by Xue et al., as depicted in Figure 8. Their antimicrobial activity was evaluated against three Gram-positive strains (*S. aureus* (4220), *S. mutans* (3289), and *S. aureus* (MRSA CCARM 3167))*,* one Gram-negative strain (*E. coli* (1924)), and one fungus (*C. albicans* (7535)) [59]. The antimicrobial data revealed two lead compounds, carbazoles **56c** and **58a**. For instance, the most active, compound **56c,** demonstrated a strong inhibition activity (MIC values of 0.5 μg/mL against both MRSA CCARM 3167 and *E. coli*). This is four-fold superior in comparison to the standard drug gatifloxacin (MIC value of 2 μg/mL). Lastly, Zawadzka evaluated the antimicrobial activity of 4-[4-(benzylamino)butoxy]-9*H*-carbazole, drawn in Figure 8 [60]. Carbazole derivative **60** was prepared in two substitution steps from commercially available 4-hydroxycarbazole. Following standard procedures, this new adduct was examined against a large panel of Gram-positive (*S. aureus* (ATCC 29213, ATCC 25923, ATCC 6358, ATCC 700699, and ATCC 43300), *S. epidermidis* (ATCC 12228), and *S. pyogenes* (ATCC 19615)) and Gram-negative (*E. coli* (ATCC 25922), *P. hauseri* (ATCC 13315), and *P. aeruginosa* (ATCC 15442)) bacteria, as well as fungal strains (*C. albicans* (ATCC 10231), and *A. flavus* (ATCC 9643)). From this study, it was determined that fungi and Gram-negative bacteria were more resistant than Gram-positive strains, although a positive control is needed to fully assess these bacterial strains [60].

## 3. Conclusions

This review condenses the known reports of various carbazole derivatives and their antimicrobial activities, which are attractive structural motifs in synthetic organic chemistry because of their tunable electronic and steric properties. As summarized above, the presence of carbazole moieties has proven effective in enhancing the antimicrobial activity of numerous compounds. Some carbazole derivatives exhibited strong in vitro inhibitory activity towards bacteria with comparable or even superior activity when compared to the positive control drugs. Therefore, it is believed that the handy literature regarding carbazole derivatives and their associated antimicrobial activities would be greatly useful in the designing of novel, potent, and safe carbazole derivatives against microbial diseases in the near future.

## Data Availability

Not applicable.

## References

[1] Aslam B., Wang W., Arshad M.I., Khurshid M., Muzammil S., Rasool M.H., Nisar M.A., Alvi R.F., Aslam M.A., Qamar M.U. (2018). Antibiotic resistance: A rundown of a global crisis. Infect. Drug Resist..

[2] Zaman S.B., Hussain M.A., Nye R., Mehta V., Mamun K.T., Hossain N. (2017). A Review on antibiotic resistance: Alarm Bells are Ringing. Cureus.

[3] World Health Organization New Report Calls for Urgent Action to Avert Antimicrobial Resistance Crisis. https://www.who.int/news/item/29-04-2019-new-report-calls-for-urgent-action-to-avert-antimicrobial-resistance-crisis.

[4] Shinu P., Mouslem A.K.A., Nair A.B., Venugopala K.N., Attimarad M., Singh V.A., Nagaraja S., Alotaibi G., Deb P.K. (2022). Progress report: Antimicrobial drug discovery in the resistance era. Pharmaceuticals.

[5] Mitcheltree M.J., Pisipati A., Syroegin E.A., Silvestre K.J., Klepacki D., Mason J.D., Terwilliger D.W., Testolin G., Pote A.R., Wu K.J.Y. (2021). A synthetic antibiotic class overcoming bacterial multidrug resistance. Nature.

[6] Johnston S.L., Blasi F., Black P.N., Martin R.J., Farrell D.J., Nieman R.B., Investigators T. (2006). The effect of telithromycin in acute exacerbations of asthma. N. Engl. J. Med..

[7] Saravolatz L.D., Leggett J. (2003). Gatifloxacin, gemifloxacin, and moxifloxacin: The role of 3 newer fluoroquinolones. Clin. Infect. Dis..

[8] Karpecki P., Depaolis M., Hunter J.A., White E.M., Rigel L., Brunner L.S., Usner D.W., Paterno M.R., Comstock T.L. (2009). Besifloxacin ophthalmic suspension 0.6% in patients with bacterial conjunctivitis: A multicenter, prospective, randomized, doublemasked, vehicle-controlled, 5-day efficacy and safety study. Clin. Ther..

[9] Ellis-Grosse E.J., Babinchak T., Dartois N., Rose G., Loh E. (2005). The efficacy and safety of tigecycline in the treatment of skin and skin-structure infections: Results of 2 double-blind phase 3 comparison studies with vancomycin-aztreonam. Clin. Infect. Dis..

[10] Ramirez J., Dartois N., Gandjini H., Yan J.L., Korth-Bradley J., McGovern P.C. (2013). Randomized phase 2 trial to evaluate the clinical efficacy of two high-dosage tigecycline regimens versus imipenemcilastatinfor treatment of hospital-acquired pneumonia. Antimicrob. Agents Chemother..

[11] Karlowsky J.A., Nichol K., Zhanel G.G. (2015). Telavancin: Mechanisms of action, in vitro activity, and mechanisms of resistance. Clin. Infect. Dis..

[12] Alt S., Bernasconi A., Sosio M., Brunati C., Donadio S., Maffioli S.I. (2019). Toward single-peak dalbavancin analogs through biologyand chemistry. ACS Chem. Biol..

[13] Nicholson S.C., Welte T., File T.M., Strauss R.S., Michiels B., Kaul P., Balis D., Arbit D., Amsler K., Noel G.J. (2012). A randomised, double-blind trial comparing ceftobiprole medocaril with ceftriaxone with or without linezolid for the treatment of patients with community-acquired pneumonia requiring hospitalisation. Int. J. Antimicrob. Agents.

[14] Lovering A.L., Gretes M.C., Safadi S.S., Danel F., de Castro L., Page M.G.P., Strynadka N.C.J. (2012). Structural insights into the anti-methicillin-resistant *Staphylococcus aureus* (MRSA) activity of ceftobiprole. J. Biol. Chem..

[15] Graebe C., Glaser C. (1872). Ueber carbazol. Justus Liebigs Ann. Chem..

[16] Chakraborty D.P., Barman B.K., Bose P.K. (1965). On the constitution of murrayanine, a carbazole derivative isolated from *Murraya koenigii* Spreng. Tetrahedron.

[17] Głuszyńska A. (2015). Biological potential of carbazole derivatives. Eur. J. Med. Chem..

[18] Caruso A., Ceramella J., Iacopetta D., Saturnino C., Mauro M.V., Bruno R., Aquaro S., Sinicropi M.S. (2019). Carbazole derivatives as antiviral agents: An overview. Molecules.

[19] Caruso A., Iacopetta D., Puoci F., Rita Cappello A., Saturnino C., Stefania Sinicropi M. (2016). Carbazole derivatives: A promising scenario for breast cancer treatment. Mini Rev. Med. Chem..

[20] Knölker H.-J., Reddy K.R. (2002). Isolation and synthesis of biologically active carbazole alkaloids. Chem. Rev..

[21] Garbett N.C., Graves D.E. (2004). Extending nature’s leads: The anticancer agent ellipticine. Curr. Med. Chem. Anticancer Agents.

[22] Ruiz-Ceja K.A., Chirino Y.I. (2017). Current FDA-approved treatments for non-small cell lung cancer and potential biomarkers for its detection. Biomed. Pharmacother..

[23] Stone R.M., Manley P.W., Larson R.A., Capdeville R. (2018). Midostaurin: Its odyssey from discovery to approval for treating acute myeloid leukemia and advanced systemic mastocytosis. Blood Adv..

[24] Gutierrez L., Jang M., Zhang T., Akhtari M., Alachkar H. (2018). Midostaurin reduces regulatory T cells markers in acute myeloid leukemia. Sci. Rep..

[25] Fisher L.D. (1999). Carvedilol and the Food and Drug Administration (FDA) approval process: The FDA paradigm and reflections on hypothesis testing. Control. Clin. Trials.

[26] Méjean A., Guillaume J.-L., Strosberg A.D. (1995). Carazolol: A potent, selective β3-adrenoceptor agonist. Eur. J. Pharmacol. Mol. Pharmacol..

[27] Papich M.G. (2008). An update on nonsteroidal anti-inflammatory drugs (NSAIDs) in small animals. Vet. Clin. N. Am. Small Anim. Pract..

[28] Balbisi E. (2004). Frovatriptan succinate, a 5-HT1B/1D receptor agonist for migraine. Int. J. Clin. Pract..

[29] Ramsewak R.S., Nair M.G., Strasburg G.M., DeWitt D.L., Nitiss J.L. (1999). Biologically active carbazole alkaloids from *Murraya koenigii*. J. Agric. Food Chem..

[30] Danish I.A., Prasad K.J.R. (2003). A one pot synthesis and evaluation of 13-oxo-quino[3,4-b]carbazol-N-oxides as antimicrobial agents. Acta Pharm..

[31] Sangeetha V., Rajendra Prasad K.J. (2004). Synthesis of isoxazolo and pyrazolino annelated carbazoles from 2-(4′-methoxy)benzylidene-1-oxo-1,2,3,4-tetrahydrocarbazoles. Asian J. Chem..

[32] Vandana T., Rajendra Prasad K.J. (2005). Synthesis of 3-phenyl-4-oxopyrano[2,3-a]carbazoles (indoloisoflavones). Indian J. Chem. Sect. B.

[33] Mukhlesur Rahman M., Gray A.I. (2005). A benzoisofuranone derivative and carbazole alkaloids from *Murraya koenigii* and their antimicrobial activity. Phytochemistry.

[34] Surendiran T., Balasubramanian S., Sivaraj D. (2008). Synthesis and characterization of novel isoxazolyl and pyrazolyl 2, 3, 4, 9 tetrahydro-1H-carbazoles and their antimicrobial studies. OCAIJ.

[35] Rajakumar P., Sekar K., Shanmugaiah V., Mathivanan N. (2009). Synthesis of novel carbazole based macrocyclic amides as potential antimicrobial agents. Eur. J. Med. Chem..

[36] Gu W., Wang S. (2010). Synthesis and antimicrobial activities of novel 1H-dibenzo[a,c]carbazoles from dehydroabietic acid. Eur. J. Med. Chem..

[37] Kaplancikli Z.A. (2011). Synthesis of some novel carbazole derivatives and evaluation of their antimicrobial activity. Marmara Pharm. J..

[38] Bandgar B.P., Adsul L.K., Lonikar S.V., Chavan H.V., Shringare S.N., Patil S.A., Jalde S.S., Koti B.A., Dhole N.A., Gacche R.N. (2013). Synthesis of novel carbazole chalcones as radical scavenger, antimicrobial and cancer chemopreventive agents. J. Enzym. Inhib. Med. Chem..

[39] Lakshmi Reddy S.V., Naresh K., Raju C.N. (2013). New sulfonamide and carbamate derivatives of 4-(oxiran-2-ylmethoxy)-9H-carbazole: Synthesis, characterization, antimicrobial and antioxidant activities. Der. Pharm. Lett..

[40] Adsul L.K., Bandgar B.P., Chavan H.V., Jalde S.S., Dhakane V.D., Shirfule A.L. (2013). Synthesis and biological evaluation of novel series of aminopyrimidine derivatives as urease inhibitors and antimicrobial agents. J. Enzym. Inhib. Med. Chem..

[41] Sharma D., Kumar N., Pathak D. (2014). Synthesis, characterization and biological evaluation of some newer carbazole derivatives. J. Serb. Chem. Soc..

[42] Chakraborty B., Chakraborty S., Saha C. (2014). Antibacterial Activity of murrayaquinone A and 6-methoxy-3,7-dimethyl-2,3-dihydro-1H-carbazole-1,4(9H)-dione. Int. J. Microbiol..

[43] Gu W., Qiao C., Wang S., Hao Y., Miao T.T. (2014). Synthesis and biological evaluation of novel N-substituted 1H-dibenzo[a,c]carbazole derivatives of dehydroabietic acid as potential antimicrobial agents. Bioorganic Med. Chem. Lett..

[44] Gu W., Hao Y., Zhang G., Wang S.-F., Miao T.-T., Zhang K.-P. (2015). Synthesis, in vitro antimicrobial and cytotoxic activities of new carbazole derivatives of ursolic acid. Bioorganic Med. Chem. Lett..

[45] Yaqub G., Sadiq Z., Hamid A., Fatima A., Ijaz Z. (2017). Conventional-microwave mediated synthesis and in vitro antimicrobial activity of novel carbazole-efflux pump inhibitor hybrid antibacterials. J. Chem..

[46] Sathiya M., Guhanathan S. (2018). Electrophilic and free radical bromination of biologically active bromo derivative of [b] carbazole using NBS/H_2_SO_4_. World J. Pharm. Res..

[47] Ochung A.A., Manguro L.A.O., Owuor P.O., Jondiko I.O., Nyunja R.A., Akala H., Mwinzi P., Opiyo S.A. (2015). Bioactive carbazole alkaloids from *Alysicarpus ovalifolius* (Schumach). J. Korean Soc. Appl. Biol. Chem..

[48] Ashok D., Ravi S., Ganesh A., Lakshmi B.V., Adam S., Murthy S.D.S. (2016). Microwave-assisted synthesis and biological evaluation of carbazole-based chalcones, aurones and flavones. Med. Chem. Res..

[49] Addla D., Wen S.-Q., Gao W.-W., Maddili S.K., Zhang L., Zhou C.-H. (2016). Design, synthesis, and biological evaluation of novel carbazole aminothiazoles as potential dna-targeting antimicrobial agents. Med. Chem. Commun..

[50] Reddy P.N., Padmaja P., Reddy B.R., Rambabu G., Kumar M.P. (2016). synthesis, molecular docking, antiproliferative, and antimicrobial activity of novel pyrano[3,2-c]carbazole derivatives. Med. Chem. Res..

[51] Clausen J.D., Kjellerup L., Cohrt K.O., Hansen J.B., Dalby-Brown W., Winther A.-M.L. (2017). Elucidation of antimicrobial activity and mechanism of action by n-substituted carbazole derivatives. Bioorganic Med. Chem. Lett..

[52] Jasass R.S., Alshehrei F., Farghaly T.A. (2018). Microwave-assisted synthesis of antimicrobial agents containing carbazole and thiazole moieties. J. Heterocycl. Chem..

[53] Zhang Y., Tangadanchu V.K.R., Cheng Y., Yang R.-G., Lin J.-M., Zhou C.-H. (2018). Potential antimicrobial isopropanol-conjugated carbazole azoles as dual targeting inhibitors of *Enterococcus Faecalis*. ACS Med. Chem. Lett..

[54] Shaikh M.S., Chandrasekaran B., Palkar M.B., Kanhed A.M., Kajee A., Mlisana K.P., Singh P., Ghai M., Cleopus Mahlalela M., Karpoormath R. (2020). Synthesis and biological evaluation of novel carbazole hybrids as promising antimicrobial agents. Chem. Biodivers..

[55] Meriç Ç., Kanbur S., Baycan F. (2021). Side chain functional carbazole-fluorene electroactive polymers: Optical, electrochemical properties, antimicrobial activity and thin film morphologies. J. Appl. Polym. Sci..

[56] Lin S., Liu J., Li H., Liu Y., Chen Y., Luo J., Liu S. (2020). Development of highly potent carbazole amphiphiles as membrane-targeting antimicrobials for treating gram-positive bacterial infections. J. Med. Chem..

[57] Bordei Telehoiu A.T., Nuță D.C., Căproiu M.T., Dumitrascu F., Zarafu I., Ioniță P., Bădiceanu C.D., Avram S., Chifiriuc M.C., Bleotu C. (2020). Design, synthesis and in vitro characterization of novel antimicrobial agents based on 6-chloro-9h-carbazol derivatives and 1,3,4-oxadiazole scaffolds. Molecules.

[58] Hegden P.R., Emmanuel B.D., Beevi J., Dharan S.S. (2021). In Silico Design, synthesis and biological evaluation of novel carbazole derivatives. J. Pharm. Chem. Res..

[59] Xue Y.-J., Li M.-Y., Jin X.-J., Zheng C.-J., Piao H.-R. (2021). Design, synthesis and evaluation of carbazole derivatives as potential antimicrobial agents. J. Enzym. Inhib. Med. Chem..

[60] Zawadzka K., Felczak A., Głowacka I.E., Piotrowska D.G., Lisowska K. (2021). Evaluation of the antimicrobial potential and toxicity of a newly synthesised 4-(4-(benzylamino)butoxy)-9h-carbazole. Int. J. Mol. Sci..

